# Quorum Sensing and Cyclic di-GMP Exert Control Over Motility of *Vibrio fischeri* KB2B1

**DOI:** 10.3389/fmicb.2021.690459

**Published:** 2021-06-28

**Authors:** Courtney N. Dial, Steven J. Eichinger, Randi Foxall, Christopher J. Corcoran, Alice H. Tischler, Robert M. Bolz, Cheryl A. Whistler, Karen L. Visick

**Affiliations:** ^1^Department of Microbiology and Immunology, Loyola University Chicago, Maywood, IL, United States; ^2^Molecular, Cellular, and Biomedical Sciences, University of New Hampshire, Durham, NH, United States

**Keywords:** *Vibrio fischeri*, luminescence, quorum sensing, c-di-GMP, motility, symbiosis, *Euprymna scolopes*

## Abstract

Bacterial motility is critical for symbiotic colonization by *Vibrio fischeri* of its host, the squid *Euprymna scolopes*, facilitating movement from surface biofilms to spaces deep inside the symbiotic organ. While colonization has been studied traditionally using strain ES114, others, including KB2B1, can outcompete ES114 for colonization for a variety of reasons, including superior biofilm formation. We report here that KB2B1 also exhibits an unusual pattern of migration through a soft agar medium: whereas ES114 migrates rapidly and steadily, KB2B1 migrates slowly and then ceases migration. To better understand this phenomenon, we isolated and sequenced five motile KB2B1 suppressor mutants. One harbored a mutation in the gene for the cAMP receptor protein (*crp*); because this strain also exhibited a growth defect, it was not characterized further. Two other suppressors contained mutations in the quorum sensing pathway that controls bacterial bioluminescence in response to cell density, and two had mutations in the diguanylate cyclase (DGC) gene *VF_1200*. Subsequent analysis indicated that (1) the quorum sensing mutations shifted KB2B1 to a perceived low cell density state and (2) the high cell density state inhibited migration *via* the downstream regulator LitR. Similar to the initial point mutations, deletion of the *VF_1200* DGC gene increased migration. Consistent with the possibility that production of the second messenger c-di-GMP inhibited the motility of KB2B1, reporter-based measurements of c-di-GMP revealed that KB2B1 produced higher levels of c-di-GMP than ES114, and overproduction of a c-di-GMP phosphodiesterase promoted migration of KB2B1. Finally, we assessed the role of viscosity in controlling the quorum sensing pathway using polyvinylpyrrolidone and found that viscosity increased light production of KB2B1 but not ES114. Together, our data indicate that while the two strains share regulators in common, they differ in the specifics of the regulatory control over downstream phenotypes such as motility.

## Introduction

The symbiosis between *Vibrio fischeri* and its host, the squid *Euprymna scolopes*, is a well-established model system used to understand bacterial processes governing colonization [reviewed in Nyholm and McFall-Ngai (2004); Stabb (2006) and Bongrand and Ruby (2019)]. The symbiosis is initiated with each generation of newly hatched squid, which recruit *V. fischeri* from the seawater. Upon entering the squid’s mantle cavity, the bacteria are directed by their host’s ciliary movements to sheltered zones on the surface of the symbiotic (light) organ where they adhere and form an aggregate or symbiotic biofilm (Nyholm et al., 2000; Nawroth et al., 2017). Subsequently, *V. fischeri* cells use chemotaxis and motility to migrate into pores, through ducts and chambers that are non-permissive for growth, to ultimately reach spaces deep inside; there, they proceed to multiply to a high cell density and produce luminescence, the product of the symbiosis (Ruby and Asato, 1993; Sycuro et al., 2006). Mutants defective for motility fail to colonize squid, while those defective for luminescence exhibit a defect in the overall colonization levels within 48 h and are ultimately lost from symbiosis over time (Graf et al., 1994; Visick et al., 2000; Lupp et al., 2003; Millikan and Ruby, 2003; Lupp and Ruby, 2005; Koch et al., 2014).

**FIGURE 1:**
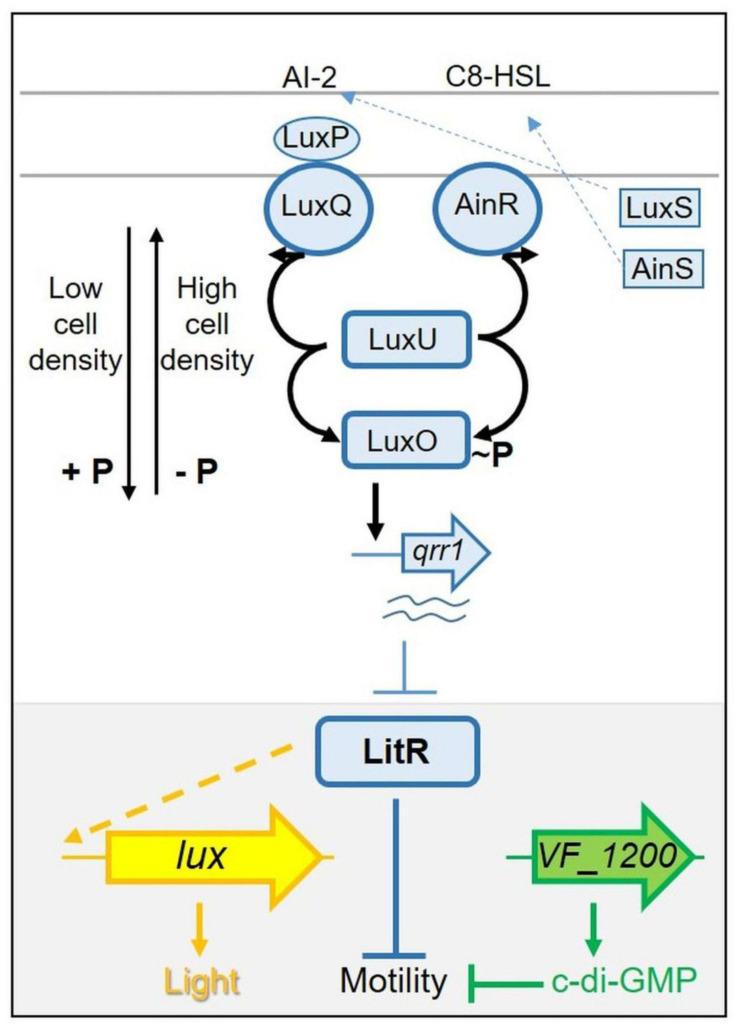
Simplified pathway(s) controlling light production and motility in *Vibrio fischeri*. LitR (blue, in gray-highlighted region at bottom of figure) is a central regulator controlling both luminescence and motility. LitR indirectly (indicated by dashed line) activates transcription of the seven gene *lux* operon (shown as a single arrow in yellow) and light production. LitR also controls motility in an as-yet-unknown way. Control of LitR production occurs, in part, *via* a complex “quorum sensing” pathway (shown at top). At low cell density, sensor kinases LuxQ and AinR function as kinases, directing the flow of phosphate *via* LuxU to LuxO, activating it to promote transcription of *qrr1*. The resulting small RNA inhibits LitR production. At high cell density, levels of the autoinducers (pheromones) AI-2 and C8-HSL build up and switch the activity of LuxQ and AinR from kinases to phosphatases, reversing the flow of phosphate and de-activating LuxO, allowing LitR to be produced. This model is based on ES114. In KB2B1, motility is also controlled by *VF_1200* (green), which encodes a diguanylate cyclase predicted to make the second messenger c-di-GMP. This small molecule is known to inhibit motility in ES114 and other bacteria.

These two phenotypes, luminescence and motility, are known to be linked in *V. fischeri*: certain regulators that control luminescence also impact motility (Lupp and Ruby, 2005; Miyashiro et al., 2010, 2014; Cao et al., 2012). The regulator at the junction of these two phenotypes is the transcription factor LitR (Fidopiastis et al., 2002). While the specific function of LitR in controlling motility is unknown, LitR controls luminescence by activating *luxR* transcription. In turn, LuxR induces the *lux* operon, resulting in light production (Figure 1). LitR itself is controlled by quorum sensing and is activated in response to increasing levels of autoinducers (AIs; also known as pheromones) [reviewed in Verma and Miyashiro (2013)]. The quorum sensing pathway includes three AI synthases, two of which, LuxS and AinS, produce AIs that control the activities of the sensor kinase/phosphatase proteins LuxQ (*via* the periplasmic protein LuxP) and AinR, respectively (Figure 1). When levels of the AIs are low, LuxQ and AinR function as kinases, resulting in phosphorylation and activation of the downstream response regulator LuxO. Phospho-LuxO activates transcription of the gene for the small RNA (sRNA) Qrr1, which in turn inhibits light production post-transcriptionally by inhibiting synthesis of LitR. As AI concentrations build, LuxQ and AinR switch their activities to phosphatases, deactivating LuxO and permitting production of LitR and in turn increasing LuxR and light levels while decreasing motility. Disruption of *litR* or its positive regulator *ainS* results in increased motility, while disruption of *luxO* results in decreased motility (Lupp and Ruby, 2005).

Historically, most work studying the symbiosis has used strain ES114, but more recently, other isolates have been described (Wollenberg and Ruby, 2009; Bongrand et al., 2016; Speare et al., 2018; Bultman et al., 2019). Some of these, including the strain KB2B1, outcompete ES114 for squid colonization (Bongrand et al., 2016). Given the importance of motility in squid colonization, here, we investigated motility of KB2B1 relative to ES114. We found that KB2B1 exhibited a substantially altered pattern of migration through soft agar. To understand the basis for this phenomenon, we isolated and sequenced motile suppressor mutants of KB2B1. We evaluated the resulting strains and found that two contained mutations in genes in the quorum sensing pathway. Two others contained mutations in a gene for a putative diguanylate cyclase (DGC), predicted to produce the second messenger cyclic di-GMP (c-di-GMP), a molecule that inhibits motility in ES114 (O’Shea et al., 2006) and other organisms (Jenal et al., 2017). Together, our studies revealed that while KB2B1 shares regulators and regulatory mechanisms in common with ES114, it also appears to exert control over downstream processes such as motility in a distinct manner.

## Materials and Methods

### Strains and Media

*Vibrio fischeri* strains used in this study are shown in Table 1. Plasmids and primers used are shown in Tables 2, 3, respectively. Wild-type strains ES114 (Boettcher and Ruby, 1990) and KB2B1 (Wollenberg and Ruby, 2009) were the parent strains used in this study. *Escherichia coli* strains were grown in Luria broth (LB) (1% tryptone, 0.5% yeast extract, and 1% sodium chloride; Davis et al., 1980). *V. fischeri* strains were routinely cultured in LBS medium (1% tryptone, 0.5% yeast extract, 2% sodium chloride, and 50 mM of Tris, pH 7.5; Graf et al., 1994; Stabb et al., 2001) at 28°C. For motility experiments, cells were grown in LBS broth or in TBS broth (1% tryptone and 2% NaCl; DeLoney et al., 2003; O’Shea et al., 2005) and inoculated onto TBS soft agar plates that were solidified with 0.25% agar and supplemented with 35 mM of MgSO_4_. For luminescence experiments, cells were grown overnight in SWT [0.5% tryptone, 0.3% yeast extract, and 70% artificial seawater (approximately 35 mM of magnesium sulfate, 7 mM of calcium chloride, 7 mM of potassium chloride, and 210 mM of sodium chloride); Yip et al., 2005] then subcultured into SWTO (SWT containing an additional 2% sodium chloride; Bose et al., 2007). For transformations, Tris-minimal medium (TMM) (Christensen and Visick, 2020) was used. Polyvinylpyrrolidone (PVP) was used in a subset of luminescence experiments to increase viscosity and had an average molecular weight of ∼55,000 (MilliporeSigma, Burlington, MA, United States). As needed, agar was added to a final concentration of 1.5% to solidify media. Antibiotics were included as appropriate at the following final concentrations: for *V. fischeri*, erythromycin (Erm), 5 μg ml^–1^; trimethoprim (Trim), 10 μg ml^–1^; kanamycin (Kan), 100 μg ml^–1^; chloramphenicol (Cm), 5 μg ml^–1^; and gentamycin (Gent), 10 μg ml^–1^; and for *E. coli*, Cm, 12.5 μg ml^–1^; Kan, 50 μg ml^–1^; and Gent, 10 μg ml^–1^.

**TABLE 1:** Strains used in this study.

Strain	Genotype	Construction^1^	References
ES114	Wild type	N/A	Boettcher and Ruby, 1990
JB19	ES114 *litR*:erm	N/A	Bose et al., 2007
KB2B1	Wild type	N/A	Wollenberg and Ruby, 2009
KV8961	KB2B1 *luxQ*-E74K (GAA to AAA)	Motile suppressor	This study
KV8962	KB2B1 *VF_1200*-fs Δ1 bp (341/2,076 nt)	Motile suppressor	This study
KV8963	KB2B1 *crp*-G185C	Motile suppressor	This study
KV8964	KB2B1 *luxO*-V106G (GTT to GGT)	Motile suppressor	This study
KV8965	KB2B1 *VF_1200*-fs Δ1 bp (1678/2,076 nt)	Motile suppressor	This study
KV8973	KB2B1 Δ*VF_1200*:FRT-Cm	TT KB2B1 with gES114 Δ*VF_1200*:FRT-Cm^2^	This study
KV9019	KB2B1 *luxO*:pAIA3	TT KB2B1 with gKV2191 (Hussa et al., 2007)	This study
KV9020	KB2B1 *luxQ*:Tn*5*	TT KB2B1 with gKV4432 (Ray and Visick, 2012)	This study
KV9023	KB2B1 *litR*:erm	TT KB2B1 with gJB19 (Bose et al., 2007)	This study
KV9067	KB2B1 Δ*VF_1200*:FRT-Cm *litR*:erm	TT KV8973 with gJB19 (Bose et al., 2007)	This study
KV9105	KB2B1 Δ*qrr1*:FRT-Erm	TT KB2B1 with gKV8867 (Tepavčević et al., In preparation)	This study
KV9115	KB2B1 Δ*VF_1200*:FRT-Cm Δ*qrr1*:FRT-Erm	TT KV8973 with gKV8867 (Tepavčević et al., In preparation)	This study

*^1^TT, TfoX-mediated transformation as described in the section “Materials and Methods”. ^2^This strain, KV8932, was made using PCR SOEing as described in Visick et al. (2018) with primer sets (templates) as follows: 2577 and 2578 (ES114), 2089 and 2090 (pKV495), and 2579 and 2580 (ES114).*

**TABLE 2:** Plasmids used in this study.

Plasmid	Description	References
pJJC4	*tfoX* + *litR* expression plasmid (Cm^R^)	Cohen, Eichinger et al., 2021
pKV302	pKV69 + *VF_0087*, engineered using primers 944 and 945	This study
pKV69	Vector control	Visick and Skoufos, 2001
pKV495	Cm^*R*^ template	Visick et al., 2018
plostfoX	*tfoX* expression plasmid (Cm^R^)	Pollack-Berti et al., 2010
plostfoX-Kan	*tfoX* expression plasmid (Kan^R^)	Brooks et al., 2014
pFY4535	c-di-GMP biosensor plasmid	Zamorano-Sanchez et al., 2019
pVAR48	pVSV105 + *luxQ*	Miyashiro et al., 2014
pVAR50	pVSV105 + *luxQ*-A216P (K + /P−)	Miyashiro et al., 2014
pVAR51	pVSV105 + *luxQ*-H378A (K−/P +)	Miyashiro et al., 2014
pVSV105	Stable expression vector (Cm^R^)	Dunn et al., 2006

**TABLE 3:** Primers used in this study.

Name	Sequence	Purpose
945	CGCATACGTTTCTACCGTTTC	Overexpress *VF_0087*
946	CCAAGCTGAACGAAGTGGAC	Overexpress *VF_0087*
2089	CCATACTTAGTGCGGCCGCCTA	Amplify antibiotic resistance cassette
2090	CCATGGCCTTCTAGGCCTATCC	Amplify antibiotic resistance cassette
2577	TAAAGCAGCAGCTGAGCAAG	Amplify sequences upstream of *VF_1200*
2578	TAGGCGGCCGCACTAAGTATGGTC GTACTTGTTCGAGCAGTAAC	Amplify sequences upstream of *VF_1200*
2579	GGATAGGCCTAGAAGGCCATGGT TAGCGCATATCAAAGAAACCATG	Amplify sequences downstream of *VF_1200*
2580	GTTTGGTGAGTACCAATCGC	Amplify sequences downstream of *VF_1200*
443	CGGTAATACTCCATAAGTTCTTTCAC	Confirm Δ*sypQ*:FRT-Cm
1189	TATTCATCTAGAGTCAGATACC	Confirm Δ*sypQ*:FRT-Cm
2577	TAAAGCAGCAGCTGAGCAAG	Confirm Δ*VF_1200*:FRT-Cm
2580	GTTTGGTGAGTACCAATCGC	Confirm Δ*VF_1200*:FRT-Cm
2425	CCTATTGCAGGGAGCGTGCCAAC	Confirm *luxO*:pAIA3
71	TTATCTTTCATTCCAAGACTGTAG	Confirm *luxO*:pAIA3
908	GCACTGAGAAGCCCTTAGAGCC	Confirm *luxQ*:Tn*5*
1304	Gatattgctttaggtgctattgatg	Confirm *luxQ*:Tn*5*
2531	Ggtaccgattaaggaagagctgttaac	Confirm *litR*:Erm
2532	Ggtaccgctgcggaagtatttgaagg	Confirm *litR*:Erm

### Strain Construction

Mutants of KB2B1 were generated as follows or as described in the *Isolation of KB2B1 motility suppressors* section: mutations generated in ES114 were transferred into KB2B1 using TfoX-mediated transformation (Pollack-Berti et al., 2010; Brooks et al., 2014; Christensen et al., 2020b; Cohen, Eichinger et al., 2021). The ES114-derived mutations were previously published as indicated in Table 1, except Δ*VF_1200*:FRT-Cm. For Δ*VF_1200*:FRT-Cm, the mutation was made in ES114 using the method of Visick et al. (2018). Briefly, PCR SOE (Splicing by Overlap Extension; Horton et al., 1989) was used to amplify and fuse ∼500-bp segments upstream and downstream of *VF_1200* with an internal Cm^R^ resistance cassette. The fused product was amplified and used to transform ES114 that carried TfoX-overproducing plasmid plostfoX-Kan (Brooks et al., 2014), selecting for cells that had recombined the Cm^R^ gene into the chromosome in place of *VF_1200*, generating KV8932. The allelic replacement was confirmed by PCR with outside primers (Table 3).

The ES114-derived mutations were transferred by transformation into KB2B1 containing a TfoX-overproducing plasmid such as plostfoX (Pollack-Berti et al., 2010) or pJJC4 (Table 2). Briefly, cells were grown in TMM (containing the appropriate antibiotic to select for the *tfoX* plasmid) at 28°C overnight with shaking, then subcultured into fresh medium, and grown at 24°C as described (Christensen et al., 2020b). These cells were then incubated with genomic DNA isolated from specific mutant strains of ES114, as described in Table 1, for 30 min, then recovered in LBS broth, and grown at 28°C for at least 90 min. The mutation of interest was selected using the appropriate antibiotic and confirmed using PCR.

### Isolation of KB2B1 Motility Suppressors

*Vibrio fischeri* strain KB2B1 was grown overnight in LBS at 28°C with shaking (225 rpm). The culture was then subcultured 1:100 into fresh LBS and grown for 2 h at 28°C with shaking. The optical density at 600 nm (OD_600_) of the culture was measured, and the cells were diluted to an OD_600_ of 0.2 in LBS before being spotted in 10 μl aliquots on TBS-Mg motility agar plates. Plates were incubated at 28°C until migration ceased, and suppressor mutants appeared at the outside edge of the zone of migration. One suppressor was harvested per motility plate with a toothpick and streaked onto LBS agar plates, which were then incubated at 28°C overnight. Single colonies were selected and grown overnight in LBS broth at 28°C. Possible suppressors were subjected to a migration assay as before to confirm their increased motility relative to the parent KB2B1 strain.

### Sequencing and Sequence Analysis

To identify mutations underlying suppressor phenotypes of KB2B1, the parental strain and its derivatives were submitted to SNPsaurus^1^ for Illumina sequencing. Putative mutations in the genomes of KB2B1 motility suppressors (KV8961–KV8965) were identified by mapping the Illumina paired-end fastq reads for each genome to the KB2B1 genome (PacBio genome polished using Illumina short reads) using Breseq version 0.33.2 (Deatherage and Barrick, 2014). The tabular summary.html output file for each of the five comparisons identified biologically relevant polymorphisms predicted in the KB2B1 derivatives. The mutation predictions were accompanied by the Illumina sequence evidence for mutation, annotation, and gene affected by each predicted mutation. Although additional potential large insertions and deletions were investigated in the analysis using statistics for sequence coverage for insertions and coverage on each side of the gap for deletions, no deletions or insertions with significant evidence were identified in the comparisons.

### Motility Assessments

Bacteria were grown overnight at 28°C in TBS and then subcultured in the same conditions to an OD_600_ between 0.2 and 0.4 (log phase). Then, cultures were normalized to an OD of 0.2, and 10 μl aliquots were spotted onto soft agar motility plates (TBS-Mg) and incubated at 28°C for 6–24 h. Spot diameter measurements were taken at the times indicated in the figures. For pictures, the plates were illuminated from below, and images were captured with an iPhone camera.

### Luminescence Experiments

Cells were grown overnight at 28°C in SWT medium. The strains were subcultured to an OD_600_ of 0.05 in SWTO and grown with shaking at 24°C. For experiments that incorporated PVP, this reagent was added to a final concentration of 3.5% in SWTO after autoclaving. Aliquots (1 ml) of cultures were taken at intervals (30 min to 1 h) for luminescence (using a GloMax^®^ 20/20 Luminometer; Promega, Madison, WI, United States) and OD_600_ (using a Fisher Biophotometer) measurements. For accuracy, measurements were taken using diluted samples when the cultures reached an OD_600_ above 1. Specific luminescence, which estimates the amount of light per cell, was calculated by dividing the amount of relative light units produced by 1 ml of culture by the OD_600_ measurement of that culture. Specific luminescence was plotted against the OD_600_ of the culture to permit comparison of the strains at the same cell density. Because luminescence experiments are performed and normalized as described above, the resulting data exhibit both variable X and Y values (for example, no two strains, or even duplicates, have the exact same OD_600_), making statistical measurements infeasible for evaluations of luminescence over a growth curve. As a result, each experiment was performed using duplicate cultures at least two times, and a representative experiment with both replicates is shown to demonstrate the consistency of the results.

### Cyclic di-GMP Biosensor Assay

Relative levels of c-di-GMP were estimated using the c-di-GMP biosensor plasmid pFY4535 (Zamorano-Sanchez et al., 2019). This plasmid contains the gene for red fluorescent protein (RFP) under the control of a c-di-GMP-binding riboswitch. It also contains the gene for AmCyan protein, expressed independently of c-di-GMP. Increasing levels of c-di-GMP result in increased production of RFP and thus increased ratios of RFP to AmCyan. For liquid measurements, strains carrying pFY4535 were grown in Gent-containing LBS medium with shaking overnight at 28°C and then subcultured for 16–24 h under the same conditions. Liquid cultures were concentrated by centrifugation and resuspended in 1 ml of phosphate-buffered saline (PBS). The samples were then washed three times with PBS, and 1 μl of the washed samples was added to 1 ml of PBS. These diluted samples were evaluated for production of RFP and green fluorescent protein (GFP) using the LSRFortessa flow cytometer (BD Biosciences, San Jose, CA, United States) using AmCyan and PE-TexasRed channels, and the data were analyzed *via* FlowJo software (Ashland, OR, United States). The resulting data were first gated on live cells and then for AmCyan and RFP double-positive cells. The resulting populations were depicted using a representative histogram of PE-TexasRed (RFP) levels to highlight relative differences in c-di-GMP. The y-axis was normalized to mode to account for differences in event count of the samples. The geometric mean fluorescence intensities of the PE-TexasRed curves were quantified and analyzed in the accompanying graphs.

### Statistics

All error bars shown represent the standard deviation. Prism 6 (GraphPad, San Diego, CA, United States) was used to generate graphs and perform statistical analysis. One-way or two-way ANOVAs and unpaired *t*-tests were used to analyze data for each graph as noted. For two-way ANOVA analyses, Šídák’s multiple comparisons test was used, where the independent variable was time (h) and the dependent variable was diameter (mm).

## Results

### Motility of KB2B1 Differs From That of ES114

**FIGURE 2:**
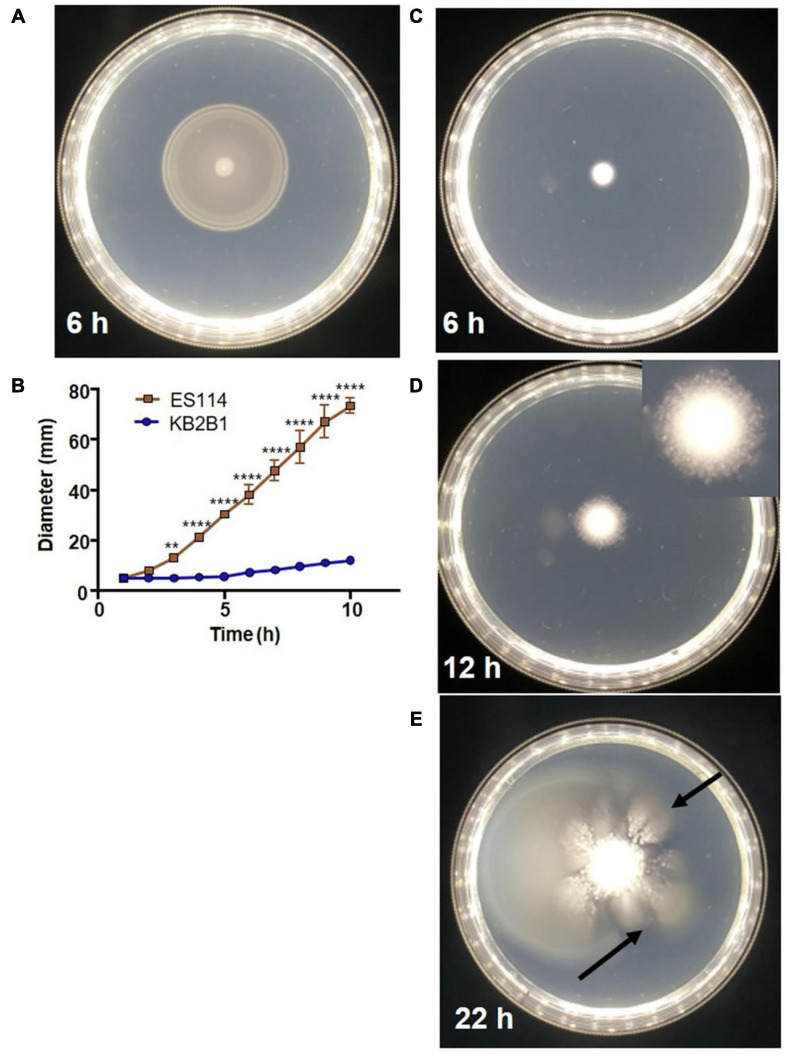
Motility of wild-type *Vibrio fischeri* strains. Motility on TBS-Mg^2+^ soft agar of strains ES114 **(A)** (6 h) and KB2B1 (6 h) **(C)**, 12 h **(D)**, and 22 h **(E)**. **(B)** The measurements of the size of the diameter of the migrating cells over time. **(D)** Inset image is cropped and magnified 1.5 × to show the appearance of the puncta. **(E)** Arrows point to suppressor mutants. Photos are representative of typical experiments. Statistics were done using a two-way ANOVA. **p = 0.0074; ****p < 0.0001.

**FIGURE 3:**
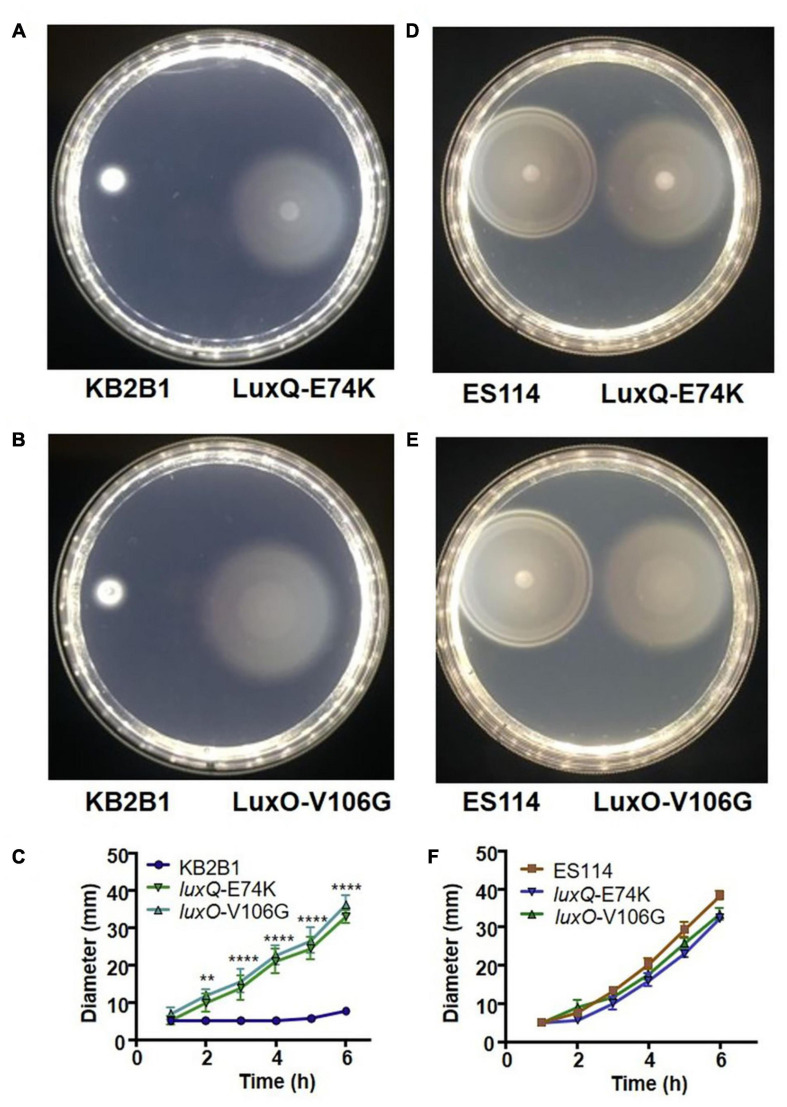
Motility of KB2B1 point mutant suppressors. Motility on TBS-Mg^2+^ agar of KB2B1 with motility suppressors **(A)** LuxQ-E74K (KV8961) and **(B)** LuxO-V106G (KV8964). Photos were taken 6 h after inoculation. **(C)** Measurements of the size of the diameter of the migrating strains (KB2B1, KV8961, and KV8964) over time. **(D,E)** Motility of the KB2B1 motility suppressor mutants LuxQ-E74K **(D)** and LuxO-V106G **(E)** compared with ES114. **(F)** Measurements of the size of the diameter of the migrating strains (ES114, KV8961, and KV8964) over time. Statistics were done using a two-way ANOVA. **p = 0.002; ****p < 0.0001.

In comparative analyses that sought to identify *in vitro* phenotypic differences between the well-characterized wild-type squid symbiont ES114 and the recently isolated colonization-dominant strain KB2B1, we found that the latter strain exhibited unusual migration on soft agar medium (Figure 2 and Supplementary Videos 1, 2). ES114 migrates steadily through the soft agar, nearing the edges of the petri plate within 10 h (Figures 2A,B). In contrast, KB2B1 migrated slowly, covering an estimated tenth of the plate within the same time frame (Figures 2B,C). Moreover, after about 12 h, KB2B1 aborted swimming, and the cells formed puncta (Figure 2D; see inset and Supplementary Video 2). Although the basis for these puncta is undetermined, the puncta do not appear to represent biofilms: disruption of neither of two different polysaccharide loci [*syp* nor *bcs* (cellulose)] (Yip et al., 2005; Bassis and Visick, 2010) nor of the gene for surface adhesin LapV (Christensen et al., 2020a) altered their appearance (Supplementary Figure 1). Finally, with prolonged incubation (within 22 h), “flares” of migrating cells were readily observed (arrows in Figure 2E). We hypothesized that these motile cells arose due to suppressor mutations that restored swimming to KB2B1. To determine if these motile cells differed from their parent, the cells from individual “flares” were isolated and re-grown, and their motility was assessed. Indeed, the resulting variants had migration patterns that more closely matched those of ES114 (Figure 3). Together, these observations indicate that KB2B1 controls its motility behavior differently than ES114.

### Quorum Sensing Genes Control KB2B1 Motility

To begin to understand the pathways that control the slow migration and punctum formation by KB2B1, we sequenced the genomes of five motile variants to identify suppressor mutations (Table 4). The five strain variants each contained distinct mutations. One of the motility suppressors acquired a mutation in *crp* (encoding cAMP receptor protein) (Lyell et al., 2013), two acquired mutations in quorum sensing regulators, and two acquired mutations in a putative DGC gene. Because the *crp* mutation impaired growth (data not shown), we did not pursue characterization of this variant further.

**TABLE 4:** Identity of KB2B1 motility suppressor mutants.

Strain	Gene mutated	Nature of mutation
KV8961	*luxQ*	E74K (GAA→AAA)
KV8962	*VF_1200*	Frameshift Δ − 1 bp at nt 341 (341/2,076 nt)
KV8963	*crp*	G185C (GGC→TGC)
KV8964	*luxO*	V114G (V106G) (GTT→GGT)
KV8965	*VF_1200*	Frameshift Δ − 1 bp at nt 1,678 (1,678/2,076 nt)

The two quorum sensing mutants contained point mutations in *luxQ* (encoding LuxQ-E74K) and *luxO* (encoding LuxO-V106G), respectively. In contrast to KB2B1, which exhibited minimal migration, the suppressor mutants exhibited detectable migration within 2 h and continued to migrate over the 6 h time course (Figures 3A–C). Indeed, the migration rates of both the *luxQ* and *luxO* suppressors were largely comparable with those of ES114 (Figures 3D–F). The major difference between ES114 and the quorum-sensing suppressor mutants was in the pattern of migration: the *luxQ* and *luxO* suppressors exhibited non-distinct rings and overall had a “fuzzier” appearance when compared with ES114 (Figures 3D,E).

The mutations in *luxQ* and *luxO* could either decrease/abolish protein function or increase activity. Past work in ES114 demonstrated that components of the quorum sensing pathway can modulate motility (e.g., Lupp and Ruby, 2005; Miyashiro et al., 2014). Specifically, null mutations in *luxO* decrease migration in soft agar. Because the KB2B1 mutant exhibits increased motility, rather than decreased motility, we considered the possibility that the *luxO* point mutation results in constitutive LuxO (i.e., LuxO∼P) activity (e.g., mimicking low cell density conditions; Figure 1). Indeed, the same mutation (encoding LuxO-V106G) was recently reported as an increased activity allele in ES114 (Kimbrough and Stabb, 2015). We tested this hypothesis and the corollary for the *luxQ* suppressor mutant using two complementary approaches. First, we evaluated luminescence of the suppressors and newly engineered *luxO* and *luxQ* null mutants. Second, we evaluated motility of the null allele strains.

**FIGURE 4:**
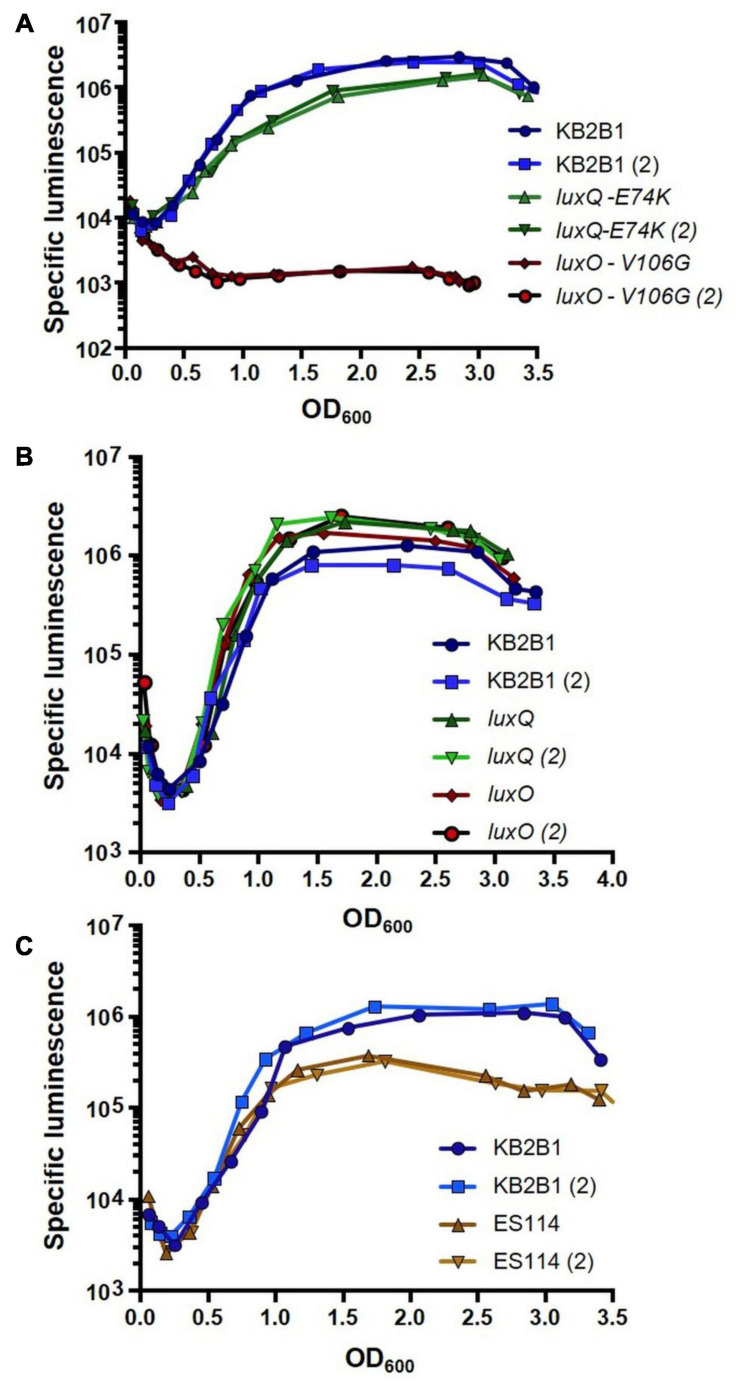
Luminescence of *Vibrio fischeri* wild-type and *lux* mutant strains. Shown are the levels of specific luminescence (relative light units divided by optical density) graphed vs. optical density (OD_600_). **(A)** KB2B1 and motility suppressor mutants KV8961 (LuxQ-E74K) and KV8964 (LuxO-V106G). **(B)** KB2B1 and *lux* null mutants KV9020 (*luxQ*) and KV9019 (*luxO*). **(C)** KB2B1 and ES114. Experiments were performed with duplicate cultures.

We expected that if the suppressor mutations caused increased activity of LuxO/LuxQ (increased LuxQ kinase activity and/or mimicking LuxO∼P), then they should decrease the luminescence of KB2B1. Indeed, both suppressor mutants produced less luminescence relative to their parent (Figure 4A). Despite the similar impact of these mutations on bacterial migration in soft agar (Figures 3D–F), the *luxO* mutation exerted a much greater impact on luminescence than did the *luxQ* mutation (>10-fold decreased specific luminescence for the *luxO* point mutant relative to ∼2-fold for the *luxQ* mutant). Engineered null mutations modestly increased luminescence in KB2B1 (Figure 4B). Finally, a comparison of the two wild-type strains revealed that KB2B1 consistently produced light levels that were modestly higher than ES114 (about threefold higher) (Figure 4C). Together, these results suggest that, under the conditions tested, the quorum sensing pathways in the respective strains function similarly.

An examination of the migration patterns of the *luxO* and *luxQ* null mutants revealed a modest decrease in motility of both, with delayed migration and a slight decrease in the diameter of the center spot at 16 h (Supplementary Figures 2A–D). In addition, the *luxO* null mutant appeared to have fewer puncta, and fewer suppressors arose in this derivative relative to KB2B1, although the number of assumed suppressors (puncta) was not quantified relative to wild type (Supplementary Figures 2E–G). These data support the hypothesis that the *lux* point mutations resulted in increased LuxO activity.

### LuxQ Phosphatase Activity Inhibits KB2B1 Motility

**FIGURE 5:**
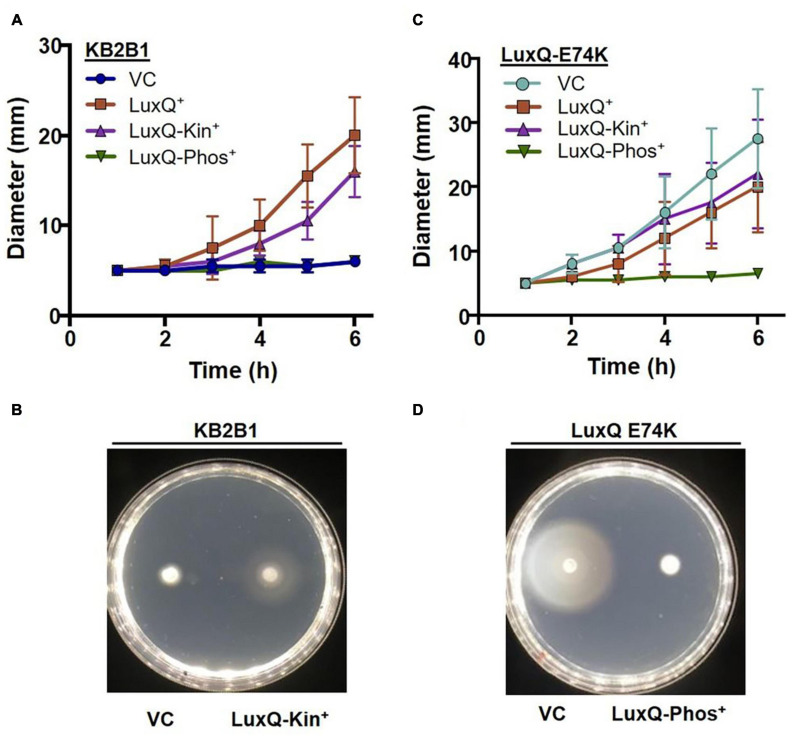
Motility of KB2B1 expressing LuxQ variants. Migration on TBS-Mg^2+^ agar of **(A,B)** KB2B1 or **(C,D)** KV8961 (*luxQ*-E74K) carrying vector control (VC) pVSV105 or plasmids expressing ES114 wild-type LuxQ (LuxQ^+^) (pVAR48) or variants of ES114 LuxQ with activity that is kinase-only (LuxQ-Kin^+^) (pVAR50) or phosphatase-only (LuxQ-Phos^+^). Time courses are shown in panels **(A,C)**, while representative images are shown in panels **(B,D)**.

To obtain additional evidence that the suppressor mutants exhibit increased motility due to the activity of the quorum sensing pathway, we modulated LuxQ activity using plasmid-based ES114 alleles that expressed either wild-type LuxQ or variants of LuxQ with kinase-only or phosphatase-only activity. Migration of the vector-control strain was poor, similar to the parent KB2B1 strain (Figure 5). The poor migration of KB2B1 was suppressed by overexpression of the ES114 LuxQ protein as well as by a LuxQ variant with constitutive kinase activity, but not by a LuxQ variant with constitutive phosphatase activity (Figures 5A,B).

We performed a similar experiment using the KB2B1 *luxQ* suppressor mutant as the parent. We found that the increased migration of this suppressor strain was substantially diminished by expression of the ES114 LuxQ variant with constitutive phosphatase activity, but not by one with constitutive kinase activity or by addition of LuxQ_ES114_ (Figures 5C,D). These data indicate that the phosphatase activity of LuxQ inhibits motility of KB2B1, while the kinase activity promotes it. Because phosphatase activity occurs at high cell density, these data indicate that KB2B1 cells may be experiencing high cell density in the motility plates and that the increased activity *lux* mutations restore cells to a low cell density, motile phenotype.

### LitR Negatively Controls Motility in KB2B1

**FIGURE 6:**
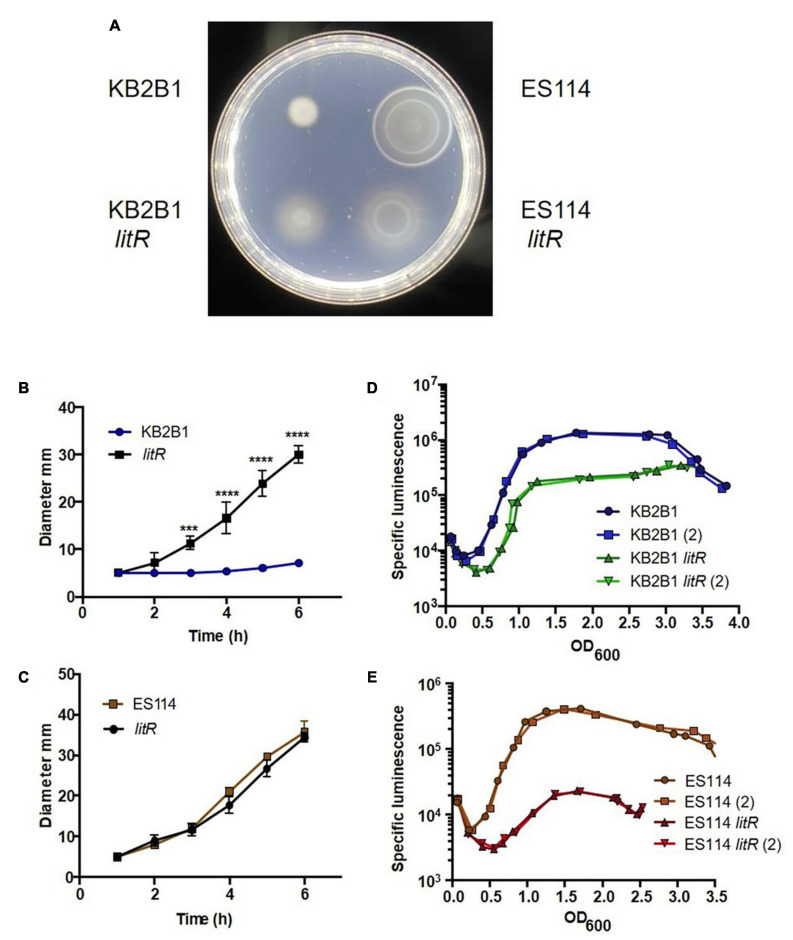
Motility and luminescence of *litR* null mutant strains. **(A)** Photograph of the motility, taken at 5 h post-inoculation, on TBS-Mg^2+^ agar of KB2B1 and its *litR* derivative (KV9023) on the left and of ES114 and its *litR* derivative (JB19) on the right. **(B,C)** Measurements of the size of the diameter of the migrating cells over time for KB2B1 and KV9023 **(B)** and ES114 and JB19 **(C)**. **(D,E)** The levels of specific luminescence (relative light units divided by optical density) graphed vs. optical density (OD_600_) of KB2B1 and its *litR* derivative (KV9023) **(D)** and ES114 and its *litR* derivative (JB19) **(E)**. Luminescence experiments were performed with duplicate cultures. Statistics were done *via* two-way ANOVA. ***p = 0.0003; ****p < 0.0001.

In ES114, LuxO indirectly controls the activity of LitR by activating transcription of Qrr1, which in turn negatively controls the levels of LitR (Miyashiro et al., 2010; Figure 1). We hypothesized that if high cell density inhibits motility due to the activity of LitR, then a *litR* null mutant would exhibit increased migration in soft agar plates. To verify the role of this downstream quorum sensing regulator in controlling motility, we engineered a null *litR* mutant of KB2B1. Indeed, the *litR* mutant migrated steadily through the soft agar, at a rate similar to that of the *lux* suppressor mutants (Figures 6A,B). As a control, we evaluated migration of the corresponding *litR* mutant of ES114. Although it has been previously reported that an ES114 *litR* mutant exhibits increased migration relative to its parent (Lupp and Ruby, 2005), we did not observe increased migration under our conditions (Figures 6A,C). Of note, the ES114 *litR* mutant exhibited a “fuzziness” in the edge of the migrating rings of cells, rather than the more compact rings formed by ES114; this phenotype has not previously been reported. This observed “fuzziness” of ES114 *litR* mutant mimics, although it does not fully match, that of the KB2B1 *litR* mutant (as well as that of the LuxQ-E74K and LuxO-V106G variants) (compare Figure 6A with Figure 3). These data lend support to the hypotheses that (i) LuxQ-E74K and LuxO-V106G impact motility *via* inhibiting LitR; and (ii) in addition to inhibiting motility, KB2B1 LitR may control the production/activity of factors necessary for formation of compact rings during migration, such as, perhaps, chemotaxis proteins, as was shown for ES114 (Bennett et al., 2020).

Further evaluation of the KB2B1 *litR* mutant revealed that luminescence was delayed and diminished, verifying that it functions in the quorum sensing pathway controlling luminescence as predicted from ES114 (Figure 6D). However, this defect was relatively modest compared with the phenotype of the ES114 *litR* mutant, which was substantial (Figure 6E), as has been previously reported (Fidopiastis et al., 2002). Overall, these data indicate that KB2B1 uses the quorum sensing pathway (Figure 1) to inhibit motility at high cell density *via* the regulator LitR. They further suggest that, under our conditions, LitR appears to play a more substantial role in inhibiting motility in KB2B1 than it does in ES114, whereas the opposite seems to be true for luminescence.

### A Diguanylate Cyclase Gene Contributes to the KB2B1 Motility Phenotype

**FIGURE 7:**
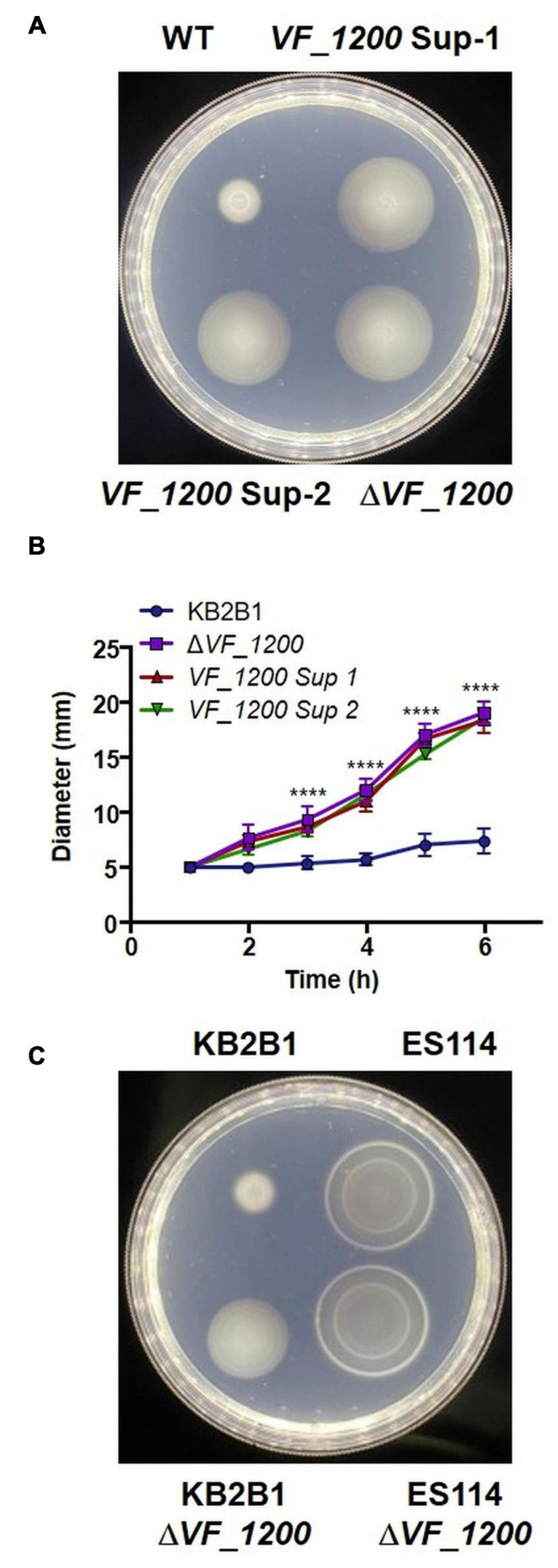
Motility of *VF_1200* mutants. **(A,B)** Migration on TBS-Mg^2+^ soft agar of wild-type KB2B1 and its derivatives strains KV8962 [*VF_1200* suppressor mutant-1 (*VF_1200*-Sup-1)], KV8965 [*VF_1200* suppressor mutant-2 (*VF_1200*-Sup-2)], and KV8973 (Δ*VF_1200*). **(A)** Captured at 6 h post-inoculation. Statistics were done *via* two-way ANOVA. ****p < 0.0001. **(C)** Migration on TBS-Mg^2+^ soft agar of wild-type KB2B1, ES114, and corresponding Δ*VF_1200* mutants (KV8973 and KV8932). **(C)** Taken at 5 h post-inoculation.

We next turned our attention to the two suppressor strains that contained mutations in a putative DGC (*VF_1200* in ES114 nomenclature) (Table 4), an enzyme that is predicted to generate c-di-GMP. In many microbes, c-di-GMP inhibits motility (Jenal et al., 2017). These mutations resulted in distinct 1-bp deletions of *VF_1200*, each resulting in a frameshift that we expected would eliminate protein function. To determine if the putative *VF_1200* null mutations were responsible for increasing motility of KB2B1, we generated a null allele by gene replacement and assessed the ability of the resulting mutant to migrate through soft agar. Whereas KB2B1 failed to migrate through soft agar, both the suppressor strains with frameshift mutations in *VF_1200* and the Δ*VF_1200* derivative migrated steadily (Figure 7). The Δ*VF_1200* mutant did not, however, migrate to the same extent as the *litR* mutant (compare Figure 7B with Figure 6B). In ES114, deletion of *VF_1200* did not substantially impact migration, perhaps because this strain already exhibits proficient migration (Figure 7C). These results confirm that *VF_1200* inhibits motility of KB2B1, albeit to a lesser extent than does LitR.

### KB2B1 Produces Elevated Levels of c-di-GMP

**FIGURE 8:**
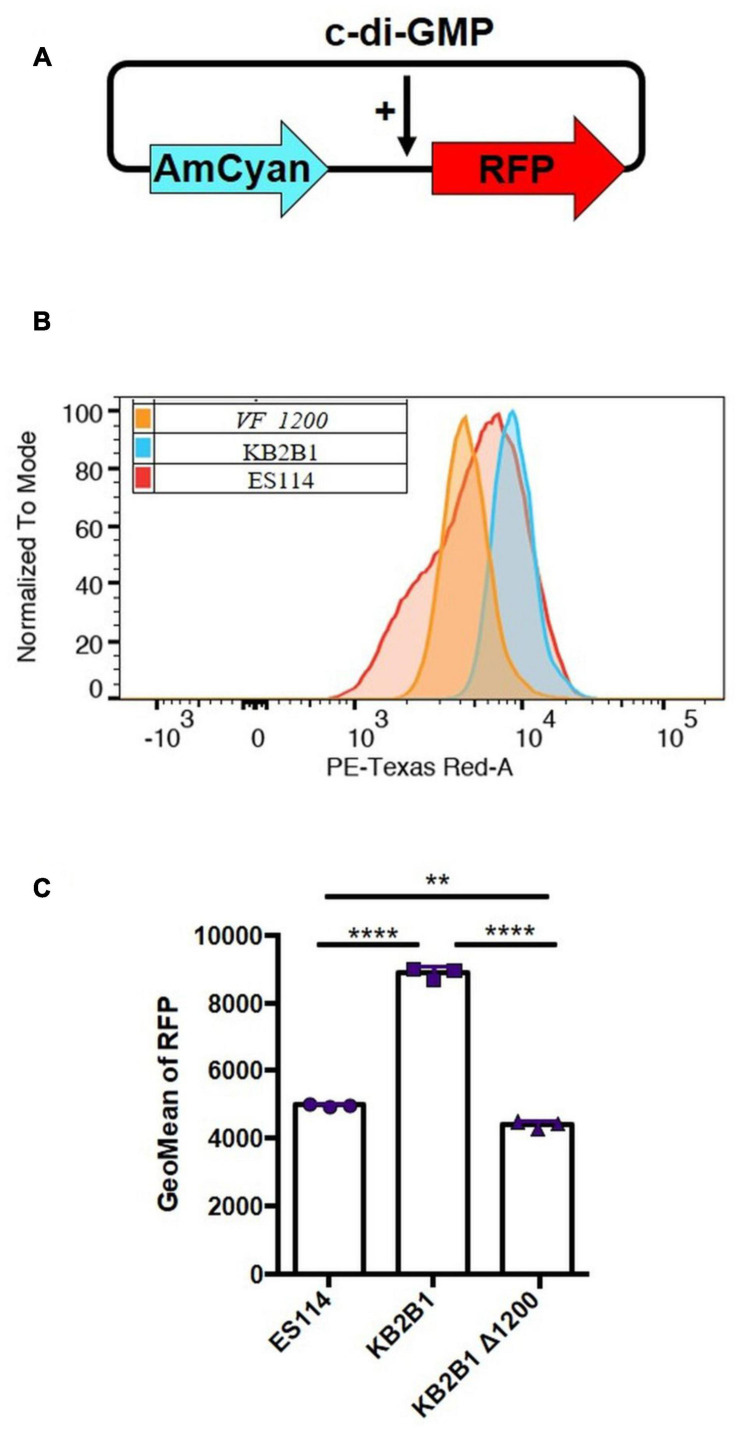
Comparisons of c-di-GMP levels. **(A)** Diagram of biosensor plasmid pFY4535 in which expression of the gene for red fluorescent protein (RFP) is controlled by a c-di-GMP riboswitch, while expression of the gene for AmCyan is independent of c-di-GMP levels. **(B,C)** Quantification of RFP relative to AmCyan following growth of pFY4535-containing KB2B1, KB2B1 Δ*VF_1200* (KV8973), and ES114 strains, as normalized to mode **(B)** and Geometric Mean (GeoMean) of RFP **(C)**. Statistics were performed using one-way ANOVA. **p = 0.0029; ****p < 0.0001.

To determine if elevated levels of c-di-GMP could be responsible for the motility phenotype of KB2B1, we first asked if the levels of c-di-GMP produced by KB2B1 differed from those produced by ES114. Using a fluorescence-based reporter of c-di-GMP (Figure 8A; Zamorano-Sanchez et al., 2019), we determined that KB2B1 produced twofold higher reporter activity relative to ES114 (Figures 8B,C). Furthermore, the levels produced by KB2B1 Δ*VF_1200* were significantly lower than those by KB2B1 (Figures 8B,C). These data suggest that increased levels of c-di-GMP contribute to the decreased motility of KB2B1.

### Overexpression of a Phosphodiesterase Increases Motility of KB2B1

**FIGURE 9:**
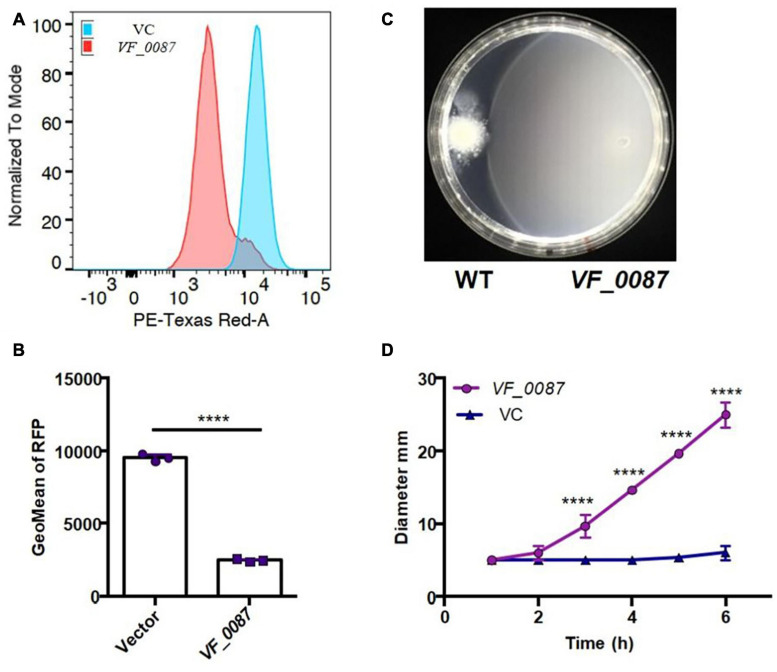
Motility of KB2B1 is increased with decreased c-di-GMP. **(A,B)** Derivatives of KB2B1 carrying pFY4535 (see Figure 8A), and either vector control plasmid pKV69 or PDE *VF_0087* expression plasmid pKV302 was analyzed for red fluorescent protein (RFP) levels relative to constitutive AmCyan signal, as normalized to mode **(A)** and GeoMean of RFP **(B)**. **(C,D)** Migration of KB2B1 containing pKV69 [wild type (WT)] or the *VF_0087*-overexpression plasmid pKV302 (*VF_0087*) on TBS-Mg^2+^ soft agar. **(C)** Captured at 12 h. Statistics in panel **(B)** were performed using an unpaired *t*-test with equal SD, and statistics in panel **(C)** were done using a two-way ANOVA. ****p < 0.0001.

To determine if high c-di-GMP levels could account for the decrease in motility of KB2B1 relative to ES114, we used plasmid pKV302 to overexpress a putative c-di-GMP phosphodiesterase, *VF_0087*, which was previously observed to impact motility of ES114 (Wolfe and Visick, 2010). Introduction of pKV302 into KB2B1 lowered the levels of c-di-GMP relative to vector-containing KB2B1 (Figures 9A,B). Importantly, expression of the phosphodiesterase increased migration of KB2B1 relative to the vector control and to about the same extent as disruption of *VF_1200* (Figures 9C,D). We conclude that motility of KB2B1 is diminished by a mechanism dependent on high levels of c-di-GMP. Of note, VF_0087-induced motility of KB2B1 resulted in migration with a compact banding pattern (Figure 9C), unlike that seen for the *litR* mutant (Figure 6A). These data lend support to the conclusion that the “fuzzy” pattern of migration observed with the *litR* mutant may be due to the loss of other regulatory activities of LitR rather than being a “default” pattern of KB2B1 migration.

### Quorum Sensing and *VF_1200* Independently Impact Motility

**FIGURE 10:**
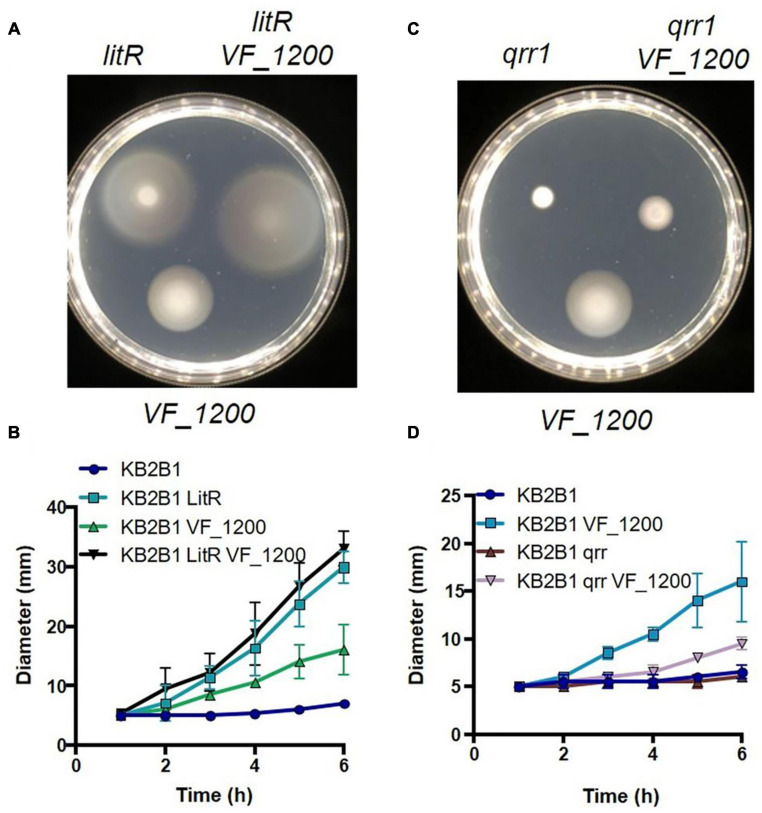
Epistasis of quorum sensing and VF_1200 pathways. Migration on TBS-Mg^2+^ soft agar of KB2B1 and derivatives **(A)** KV9023 (*litR*), KV8973 (Δ*VF_1200*), and KV9067 (*litR* Δ*VF1200*); **(B)** KV9105 (Δ*qrr1*), KV8973 (Δ*VF_1200*), and KV9115 (Δ*qrr1* Δ*VF_1200*). **(A,C)** Images were captured at 6 h. **(B,D)** Measurements of the migration on TBS-Mg^2+^ agar of the strains shown in panels **(A,C)**.

Because it was not clear if or how these pathways (quorum sensing and *VF_1200*) connected, we generated and evaluated double mutants. A Double Δ*VF_1200 litR* mutant exhibited a modest increase over migration of the *litR* single mutant (Figure 10A). Because both mutations lead to an increase in motility, making an epistatic relationship difficult to evaluate, we therefore generated and evaluated a Δ*VF_1200 qrr1* double mutant. In ES114, Qrr1 negatively regulates LitR (Figure 1), and thus a *qrr1* mutant is predicted to result in decreased motility. Indeed, the KB2B1 *qrr1* single mutant exhibited decreased migration (Figure 10B). Finally, the Δ*VF_1200 qrr1* double mutant exhibited a modest increase in migration over the single *qrr1* mutant and less migration than the Δ*VF_1200* single mutant (Figures 10C,D). Together, these data suggest that these pathways are at least partially independent of each other.

### Increased Viscosity Impacts the Quorum Sensing Pathway

Because both pathways that contribute to controlling motility of KB2B1 (quorum sensing and VF_1200-produced c-di-GMP) are conserved in ES114, neither can fully account for the difference in motility observed for the two strains. We thus considered two possibilities for the differential impact of quorum sensing on motility of KB2B1 and ES114. First, KB2B1 could sense high cell density sooner and switch into “high cell density” mode earlier than ES114. Indeed, KB2B1 produces a higher amount of luminescence than ES114, although it is not clear if this modest difference would be sufficient for the observed effect (Figure 4C). Second, KB2B1 could be more sensitive to the semi-solid medium conditions of motility agar, more readily recognizing this condition as a “surface signal” to switch from a planktonic state to a sessile state.

**FIGURE 11:**
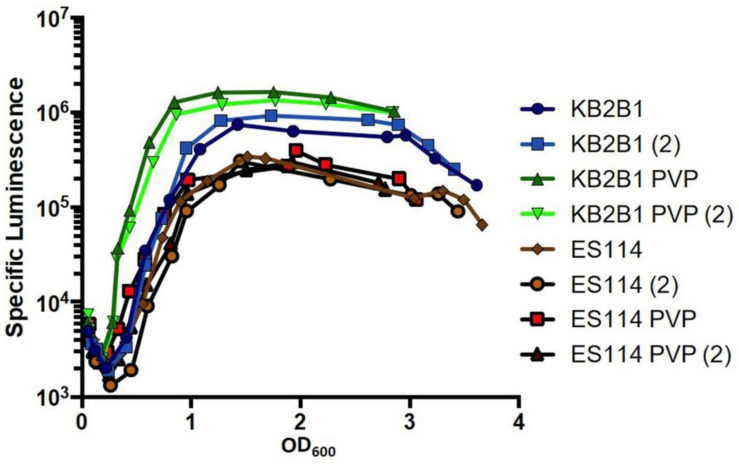
Impact of polyvinylpyrrolidone (PVP) addition on luminescence of *Vibrio fischeri*. Levels of specific luminescence (relative light units divided by optical density) graphed vs. optical density (OD_600_) of KB2B1 and ES114 grown in the absence and presence of 3.5% PVP. Luminescence experiments were performed with duplicate cultures.

To test the possibility that KB2B1 more readily recognizes surface signals and/or high viscosity, we grew KB2B1 and ES114 in a medium in which viscosity was increased using PVP (Speare et al., 2020). We predicted that KB2B1 might exhibit increased bioluminescence under these conditions, corresponding with decreased motility (Figure 1). Indeed, the addition of PVP resulted in measurably increased levels of light production by KB2B1 relative to the untreated control (Figure 11). In contrast, ES114 did not respond significantly to the change in viscosity. As a result, the addition of PVP resulted in about a sevenfold increase in luminescence of KB2B1 at low cell density. PVP also induced luminescence of a mutant defective for the flagellar component FliQ, indicating that flagella may not be necessary for relaying the viscosity signal (Supplementary Figure 3). Together, these data suggest that KB2B1 differs from ES114 in its ability to recognize and/or respond to changes in environmental viscosity, a phenomenon that may contribute to the observed difference in its ability to decrease its motility.

## Discussion

Akin to studies in *E. coli* that primarily focused on strain K-12 for years, *V. fischeri* ES114 has long been the default wild-type strain for investigators studying the *V. fischeri*–squid symbiosis, with good reason. For a relatively small field, focusing on a single wild-type strain permitted a concerted effort to develop tools and approaches for genetic manipulation, including the sequence of the genome, which became available in 2005 (Ruby et al., 2005; Mandel et al., 2008). As the field expanded, the isolation and characterization of additional strains, including the so-called “dominant” strains such as KB2B1 (Wollenberg and Ruby, 2009; Bongrand et al., 2016), along with the availability of their genomes, have broadened the scope of the work and enabled the investigation of individual variation that permits similar or even better colonization outcomes.

Like other dominant strains that have been sequenced, KB2B1 carries approximately 250 kb of additional DNA relative to ES114—not necessarily in large islands but rather in small insertions throughout the genome (Bongrand et al., 2016)—making it non-trivial to pinpoint specific genes/sequences that could be responsible for phenotypic variations. Thus, when we discovered the relative inability of KB2B1 to migrate through soft agar, we took a genetic approach, selecting for suppressor mutants, to begin to determine the underlying cause. Our discovery of roles for quorum sensing and the second messenger c-di-GMP in KB2B1 is consistent with what is known about the control of motility in *V. fischeri* and other bacteria (Fidopiastis et al., 2002; Lupp and Ruby, 2005; O’Shea et al., 2006; Jenal et al., 2017), although to date the putative DGC gene *VF_1200* has not been associated with motility control in ES114; nor does it substantially impact migration by the latter strain under the conditions used here.

The pattern produced by KB2B1 during growth and migration on soft agar is unusual, with steady movement early on, followed by an apparent slowing and cessation of migration, resulting in the formation of microcolonies or “puncta.” These puncta are somewhat reminiscent of a phenomenon in *Salmonella* known as “abortive transduction,” in which a subset of non-motile cells exposed to transducing phage carrying the missing motility factor briefly become motile (Stocker, 1956; Weinstock, 2002). These transiently motile bacteria form trails of microcolonies due to the temporary acquisition of a non-replicated, non-recombined gene or gene product that supplies the function missing in the original mutant, facilitating movement. These trails are interpreted to be the result of cell divisions in which only a single daughter cell retains the flagellum; the non-flagellated daughter cannot move and thus grows at that location to form a microcolony. We hypothesize that a similar phenomenon could occur with KB2B1, with cell divisions resulting in two daughters, one that lacks a flagellum and becomes amotile, forming a microcolony, and another that retains a flagellum and migrates a short distance before the next cell division.

Of course, we note that care must be taken when interpreting bacterial motility using migration through soft agar. In this experimental approach, bacterial migration depends not only on flagellum-mediated movement but also on other aspects of bacterial physiology, including growth, nutrient utilization, and chemotaxis to a gradient that the growing cells establish. Thus, a defect in migration could be due to any of these parameters. Indeed, we hypothesize that the “fuzzy” appearance of the migration patterns for the *litR* mutant derivatives of both ES114 and KB2B1 could be due to altered chemotaxis; Bennett et al. (2020) recently determined that LitR represses a set of four chemoreceptors in ES114. However, in our work evaluating luminescence, which takes into account growth rate, we found no evidence to support the idea that migration is severely impacted by growth rate, either in the context of wild-type KB2B1 or its derivatives. Regardless of whether the factors under investigation here impact more than flagellum-mediated motility, it is clear that they contribute to the distinct pattern of migration observed for KB2B1 relative to ES114.

When comparing the relative impact of quorum sensing and *VF_1200*, we found that the former exerted a more substantial effect on migration. Quorum sensing mutations also exerted the dominant effect in epistasis experiments: deleting *VF_1200* in *litR* and *qrr1* mutant strains only modestly increased their motility. These analyses, and the differences in the “fuzziness” of the bands of migrating bacteria, suggest that the two pathways may be distinct, or at the very least *VF_1200* is not the only factor controlling motility that functions downstream of quorum sensing.

KB2B1 encodes the same quorum sensing components as does ES114, with only minor sequence variations (Figure 1). Specifically, the KB2B1 genome includes genes for the AI synthases AinS, LuxS, and LuxI, histidine kinases AinR and LuxQ, phosphotransferase LuxU, response regulator LuxO, transcription factors LitR and LuxR, and sRNA Qrr1. Of note, in the region corresponding to the 5′ end of *ainR* in ES114, KB2B1 *ainR* contains a base change that results in a stop codon, potentially causing the absence of as many as 76 N-terminal amino acids in the resulting protein. The same base change also appears to be present in a few other *V. fischeri* strains, including MB11B1 and MB13B3. The consequence of this sequence change, and whether it contributes to the quorum sensing-influenced migration patterns observed for KB2B1, awaits further study.

Here, we hypothesized that KB2B1 could recognize a surface (such as a soft agar plate) better than ES114, resulting in (as-yet-unknown) feedback to turn off motility, at least in part through the quorum sensing pathway. The ability to sense a surface is an active area of investigation in microbiology research (Kimkeyes and Heinemann, 2020). With respect to motility, one of the best characterized systems is that of *Vibrio parahaemolyticus*. This organism switches from a polarly flagellated cell, the swimmer, to a hyperflagellated swarmer cell to swim across and eventually colonize more viscous surfaces. The switch is initiated when the polar flagellum senses that its movement is constricted by, for example, high viscosity or a surface. In conjunction with other signals, this viscosity/surface sensing induces *laf* gene expression, resulting in production of the lateral flagella necessary for swarming (McCarter and Silverman, 1990; Stewart et al., 1997). In support of the possibility that KB2B1 might control its gene expression in a similar manner, we determined that addition of the viscosity agent PVP increased the quorum sensing-dependent phenotype luminescence in KB2B1, but not ES114. While this type of surface-sensing is controlled by flagella in *V. parahaemolyticus*, and others, we found that a KB2B1 flagellar mutant retained the ability to induce luminescence in response to PVP. Thus, another factor must be involved. We developed two hypotheses to explain the pattern of migration exhibited by KB2B1. First, we propose that KB2B1 could sense viscosity or a surface and slow its motility, which in turn permits local accumulation of AIs, further decreasing flagella and motility until the cells become aflagellated. This type of surface sensing could be related to KB2B1 pili or curli, which have both been shown to modulate gene expression upon surface recognition in *E. coli* (Ogasawara et al., 2012; Evans and Chapman, 2014). Surface sensing could also occur through the cell body in general. Species of *Pseudomonas*, for example, can alter their gene expression based on a change in short-range Van der Waals and electrostatic forces sensed *via* the cell body (Hinsa et al., 2003; Cooley et al., 2013). Second, the viscosity signal itself could feed directly and in an unknown way through the quorum sensing system, which then shuts down motility. In ES114, disruption of the gene for AI synthase AinS (Figure 1) results in cells that have severely reduced luminescence in liquid but increased luminescence on plates (Lupp et al., 2003; Lyell et al., 2010). The cause underlying this phenotypic difference dependent on growth conditions remains undetermined; one explanation could be that the cultures grown on plates experience increased cell density and thus accumulation of other AIs potentially inhibited by AinS, while another one could be that the AinS-synthesized AI and/or the AinS-controlled pathway contributes control over surface/viscosity recognition. Regardless of the exact cause for the *ainS* mutant phenotypes, our work here represents a second example of how *V. fischeri* responds to its environment, potentially a surface or viscosity, to influence phenotypes under the control of quorum sensing. These findings provide important insight into the role of quorum sensing and provide a starting point for future investigations of the contribution of viscosity/surface sensing to *V. fischeri* physiology.

In summary, this work identifies a novel motility phenotype for *V. fischeri* strain KB2B1 and determines that KB2B1 naturally produces higher levels of c-di-GMP relative to the better-studied ES114. We determine that both quorum sensing and c-di-GMP contribute to control over motility and identify the putative DGC VF_1200 as a motility regulator in KB2B1. Finally, we find that KB2B1 appears more sensitive than E114 to viscosity and/or surface signals, a finding that may help explain the superior ability of the former organism to colonize its symbiotic host.

## Data Availability Statement

The datasets presented in this study can be found in online repositories. The names of the repository/repositories and accession number(s) can be found below: https://www.ncbi.nlm.nih.gov/, SAMN16729272; https://www.ncbi.nlm.nih.gov/, SAMN16727280; https://www.ncbi.nlm.nih.gov/, SAMN16727281; https://www.ncbi.nlm.nih.gov/, SAMN16727282; https://www.ncbi.nlm.nih.gov/, SAMN16727283; https://www.ncbi.nlm.nih.gov/, SAMN16727284.

## Author Contributions

CD, CC, and KV conceived the work. CD, SE, CC, RB, AT, RF, and KV collected, analyzed, and interpreted data. CD, CC, RF, and KV drafted the article. All authors approved the final version prior to manuscript submission.

## Conflict of Interest

The authors declare that the research was conducted in the absence of any commercial or financial relationships that could be construed as a potential conflict of interest.

## Footnotes

1 https://www.snpsaurus.com/
