# Supplementary material for: Quorum Sensing and Cyclic di-GMP Exert Control Over Motility of Vibrio fischeri KB2B1
Source: Front Microbiol. 2021 Jun 28;12:690459. doi: 10.3389/fmicb.2021.690459 (PMC8273514; doi:10.3389/fmicb.2021.690459)
Supplement: Supplementary file 3 [file Data_Sheet_1.docx]

Supplementary Materials

Quorum sensing and c-di-GMP exert control over motility of *Vibrio fischeri* KB2B1

**Courtney N. Dial^1^, Steven J. Eichinger^1^, Randi Foxall^2^, Christopher J. Corcoran^1^, Alice H. Tischler^1^, Robert M. Bolz^1^, Cheryl A. Whistler^2^, and Karen L. Visick^1*^**

^1^Loyola University Chicago, Department of Microbiology and Immunology, Maywood, IL USA

^2^University of New Hampshire, Molecular, Cellular, and Biomedical Sciences, Durham, NH USA

*** Correspondence:**Karen Visick
[kvisick@luc.edu](mailto:kvisick@luc.edu)

# Supplementary Figures

#
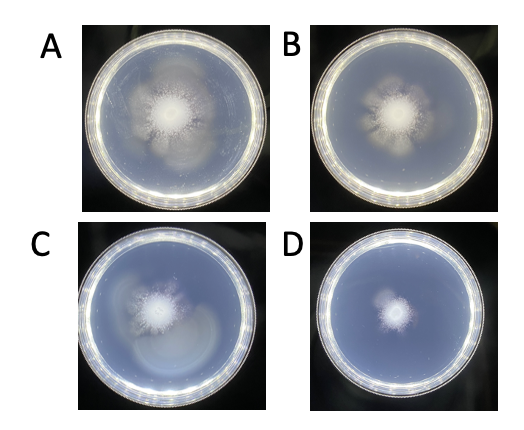


C

**Supplementary Figure 1. Motility of KB2B1 and biofilm mutant derivatives.** Motility of (A) KB2B1 and its mutant derivatives, defective for (B) *sypQ*, (C) *bcsA*, and (D) *lapV*.

**
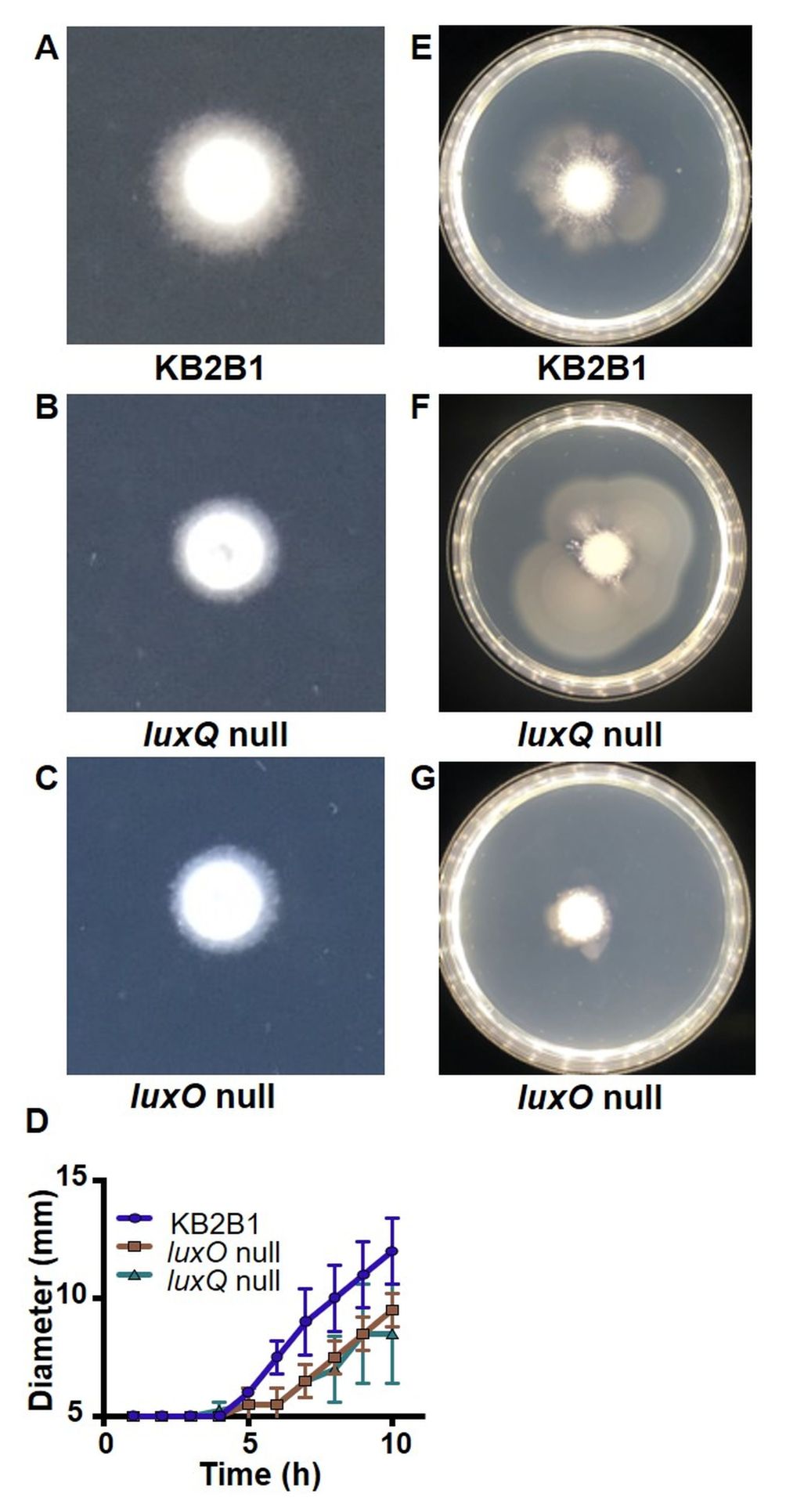
**

**Supplementary Figure 2. Motility of KB2B1 null mutants**. Migration on TBS-Mg^2+^ agar of (A) KB2B1, (B) *luxQ* null mutant (KV9020), and (C) *luxO* null mutant (KV9019). Images were captured at 6 h post-inoculation. (D) Measurements of the size of the diameter of the migrating strains over time. (E-G) Migration on TBS-Mg^2+^ agar of (E) KB2B1, (F) *luxQ* null strain (KV9020), and (G) *luxO* null strain (KV9019). Images were captured at 16 h post-inoculation.


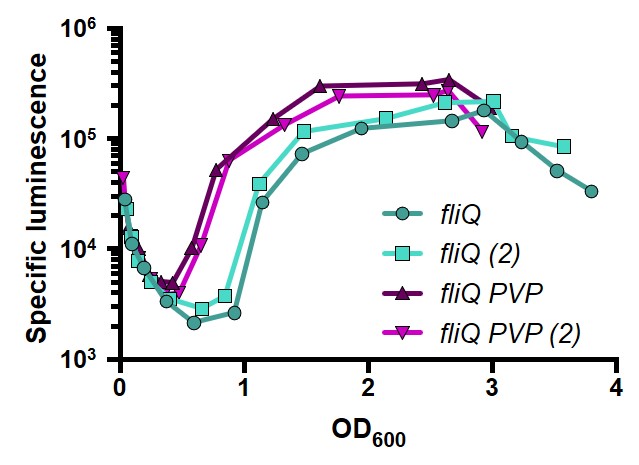


**Supplementary Figure 3. Luminescence of KB2B1 *fliQ* in the presence and absence of PVP.** Shown are the levels of specific luminescence (relative light units divided by optical density) graphed versus optical density (OD_600_) for KB2B1 *fliQ* mutant KV8636 mutant. Experiment was performed with duplicate cultures.

# Supplementary Materials and Methods

## Time-lapse videos of *V. fischeri* motility.

*V. fischeri* strains ES114 and KB2B1 were grown overnight from freezer stock in 5 ml of LBS at 28°C with shaking. The cultures were then subcultured 1:100 into fresh LBS and grown for 2 h at 28°C with shaking. The OD_600_ of the culture was measured and the cells were diluted to an OD_600_ of 0.2 in LBS before spotting 10 μl on the center of motility agar plates (1% tryptone, 2% NaCl, 0.25% agar, and 35 mM MgSO_4_). Plates were incubated at room temperature (~24°C) for 8 h for ES114, or 16 h for KB2B1 on a LED-backlit plate holder. Motility was recorded using the native time-lapse function on an Apple iPhone 6.

# Supplemental Tables

## Supplementary Table 1. Strains used in the Supplemental data section.

| KB2B1 | Wild Type | N/A^1^ | ([Wollenberg and Ruby, 2009](#_ENREF_4)) |
| --- | --- | --- | --- |
| KV8122 | KB2B1 Δ*sypQ*::FRT-Cm | TT with gKV8069 ([Tischler *et al.*, 2018](#_ENREF_2)) | This study |
| KV8636 | KB2B1 Δ*fliQ*::FRT-Trim | TT with gKV8300 ([Visick *et al.*, 2018](#_ENREF_3)) | This study |
| KV9297 | KB2B1 Δ*bcsA*::FRT-Trim | TT KB2B1 with gKV8616 ([Christensen *et al.*, 2020](#_ENREF_1)) | This study |
| KV9658 | KB2B1 *lapVtrunc*::FRT-Erm | TT KB2B1 with gKV8613 ([Christensen *et al.*, 2020](#_ENREF_1)) | This study |

^1^N/A, not applicable

## Supplemental Table 2. Primers used for work shown in the supplemental data section

| **Name** | **Sequence** | **Purpose** |
| --- | --- | --- |
| 443 | CGGTAATACTCCATAAGTTCTTTCAC | Confirm Δ*sypQ*::FRT-Cm |
| 1189 | TATTCATCTAGAGTCAGATACC | Confirm Δ*sypQ*::FRT-Cm |
| 2073 | gcatgcGTCATTTTTCGGCGAAAGAGG | Confirm Δ*bcsA*::FRT-Trim |
| 2074 | gggccctcgacataactgattgcactc | Confirm Δ*bcsA*::FRT-Trim |
| 2442 | TCGCTTGCTTCTACTTCTTTACCTTCTAGTT | Confirm *lapVtrunc*::FRT-Erm |
| 2227 | TGAAATCGCTTGAGTATCTGTAAG | Confirm *lapVtrunc*::FRT-Erm |

# References

Christensen, D. G., Marsden, A. E., Hodge-Hanson, K., Essock-Burns, T. and Visick, K. L. (2020). LapG mediates biofilm dispersal in *Vibrio fischeri* by controlling maintenance of the VCBS-containing adhesin LapV. *Mol Microbiol* 114, 742-761 DOI: 10.1111/mmi.14573.

Tischler, A. H., Lie, L., Thompson, C. M. and Visick, K. L. (2018). Discovery of calcium as a biofilm-promoting signal for *Vibrio fischeri* reveals new phenotypes and underlying regulatory complexity. *J Bacteriol* 200, e00016-00018 DOI: 10.1128/JB.00016-18.

Visick, K. L., Hodge-Hanson, K. M., Tischler, A. H., Bennett, A. K. and Mastrodomenico, V. (2018). Tools for rapid genetic engineering of *Vibrio fischeri*. *Appl Environ Microbiol* 84, e00850-00818 DOI: 10.1128/AEM.00850-18.

Wollenberg, M. S. and Ruby, E. G. (2009). Population structure of *Vibrio fischeri* within the light organs of *Euprymna scolopes* squid from Two Oahu (Hawaii) populations. *Appl Environ Microbiol* 75, 193-202 DOI: 10.1128/AEM.01792-08.

**
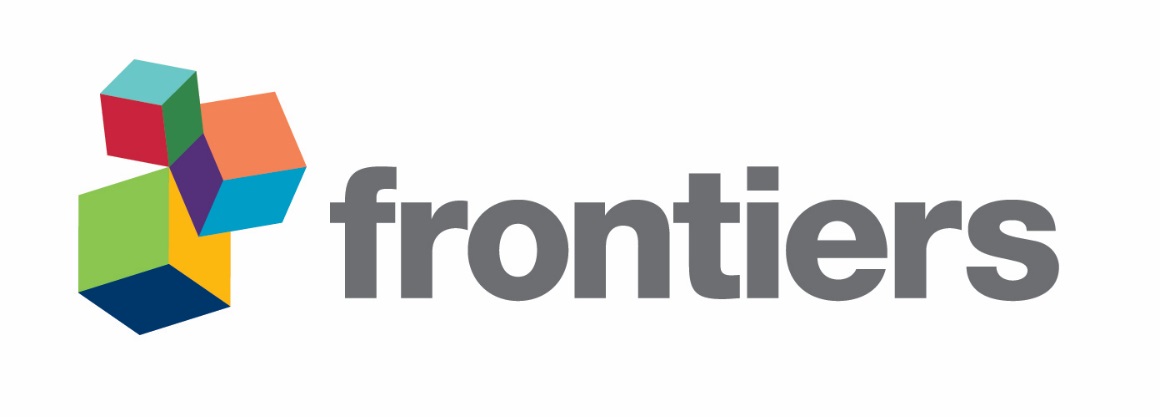
**
